# Diagnostic accuracy of a non-invasive spot-check hemoglobin meter, Masimo Rad-67® pulse CO-Oximeter®, in detection of anemia in antenatal care settings in Kenya

**DOI:** 10.3389/fgwh.2024.1427261

**Published:** 2024-10-14

**Authors:** Angela Koech, Isaac Mwaniki, Joseph Mutunga, Moses Mukhanya, Emily Mwadime, Marvine Ochieng, Grace Mwashigadi, Hiten D. Mistry, Rachel Craik, Peter von Dadelszen, Marleen Temmerman, Stanley Luchters, Geoffrey Omuse, The PRECISE Network, Patricia Okiro

**Affiliations:** ^1^ Centre of Excellence in Women and Child Health, Aga Khan University, Nairobi, Kenya; ^2^ Faculty of Medicine and Health Sciences, Ghent University, Ghent, Belgium; ^3^ Department of Obstetrics and Gynecology, Aga Khan University, Nairobi, Kenya; ^4^ Department of Women and Children’s Health, Kings College London, London, United Kingdom; ^5^ Department of International Public Health, Liverpool School of Tropical Medicine (LSTM), Liverpool, United Kingdom; ^6^ Centre for Sexual Health and HIV/AIDS Research (CeSHHAR), Harare, Zimbabwe; ^7^ Department of Pathology, Aga Khan University, Nairobi, Kenya

**Keywords:** diagnostic accuracy, non-invasive, hemoglobin, anemia, antenatal care, Kenya

## Abstract

**Background:**

Point of care hemoglobin meters play key roles in increasing access to anemia screening in antenatal care especially in settings with limited access to laboratories. We aimed to determine the diagnostic accuracy of a non-invasive spot-check hemoglobin (SpHb) meter, Masimo Rad-67® Pulse CO-Oximeter®, in the diagnosis of anemia in pregnant women attending antenatal care clinics in Kilifi, Kenya.

**Methods:**

This was a diagnostic accuracy study that retrospectively evaluated SpHb against a validated reference standard of laboratory assessed hemoglobin (Lab Hb) by a SYSMEX XN-330 automated hematology analyzer. The study was nested within a prospective pregnancy cohort study that recruited unselected pregnant women from antenatal care clinics in two public hospitals in Kilifi County, coastal Kenya. Records with both SpHb and Lab Hb were selected from pregnancy visits between May 2021 and December 2022. Linear regression and Bland-Altman analysis were performed to compare the two tests and diagnostic accuracy parameters obtained for the diagnosis of anemia.

**Results:**

A total of 2,975 records (from 2,203 unique participants), with paired SpHb and Lab Hb were analyzed. Linear regression showed a significant but weak positive correlation, a proportional bias of 0.44 (95% CI 0.41–0.47) and a constant of 7.59 (95% CI 7.30–7.87, *p* < 0.001). The median bias was 1.70 g/dl, with limits of agreement of −0.80 to 4.20. SpHb tended to be higher than Lab Hb on the low hemoglobin range but lower than Lab Hb on the high hemoglobin range. The sensitivity of SpHb in detecting anemia was 18.66%. Prevalence, specificity, positive predictive value, and negative predictive values were 46.37%, 96.77%, 83.33%, and 57.92% respectively.

**Conclusion:**

Overall, SpHb by Masimo Rad-67® Pulse CO-Oximeter® did not accurately identify pregnant women with anemia and many cases would be missed. We would not recommend its use in antenatal care settings.

## 1 Introduction

Anemia is a common problem in pregnancy that is associated with adverse maternal and perinatal outcomes, including postpartum hemorrhage, heart failure, maternal death, preterm birth, birth asphyxia, and fetal growth restriction (1–3). Anemia affects women worldwide but the largest burden is in sub-Saharan Africa and South East Asia (4, 5). In Kenya, the burden is greatest in the coastal region (6).

Both the World Health Organization (WHO) (7) and the Kenyan Ministry of Health (8) recommend routine screening for anemia as an important strategy to guide care and prevent adverse outcomes (9). The WHO recommends a full blood count (FBC) for diagnosis of anemia in pregnancy (7). However, FBC is an expensive test that utilizes a venous blood sample and requires an automated analyzer, adequate laboratory infrastructure and skilled personnel. Many health facilities in low resource settings do not have on-site laboratories (10, 11). The WHO does support the adoption of reliable low cost methods for detecting anemia where access to laboratories is limited (7). In these settings, simple point-of-care tests should be considered to provide timely results and enable prompt initiation of treatment interventions. The Masimo Rad-67® Pulse CO-Oximeter® spot-check is one such test. It is a portable and user-friendly hemoglobin meter that offers a “no-prick” assessment of hemoglobin, making it widely acceptable to both patients and providers (12, 13).

MASIMO® Pulse CO-Oximeters® have been used across various patient demographics, including adults and children (14, 15), and in diverse health care settings, including health facility (16–18) and community settings (19). While the device is not intended to measure spot-check hemoglobin (SpHb) in pregnant women (20), its features make it desirable for use in this population. The few pregnancy studies found concerns in diagnostic performance (21–23). Both had small sample sizes, and none were conducted in antenatal care settings. Such insufficient evidence to support the use of spot-check hemoglobin (SpHb) in pregnant women highlights the need to conduct a robust evaluation of its diagnostic performance in a relevant clinical setting. In our case: antenatal care clinics in low resource settings where access to laboratory based FBC is limited. There are two possible applications for SpHb in antenatal care; one as a single test to screen and treat anemia, (similar to current use of other hemoglobin assessments in antenatal care) or in a 2-step process of screening followed by a confirmatory test among those who screen positive prior to treatment initiation (24).

The objective of this study was to determine the diagnostic accuracy of a non-invasive spot-check hemoglobin (SpHb) meter, Masimo Rad-67® Pulse CO-Oximeter® against an automated analyzer (reference standard) in detecting anemia among pregnant women attending antenatal care clinics in Kilifi, Kenya. We hypothesized that the test would have a sensitivity and specificity of at least 90% and 80%, respectively.

## 2 Methodology

### 2.1 Study design

This study was a retrospective diagnostic accuracy study aiming to assess the performance of the Masimo Rad-67® Pulse CO-Oximeter® (Masimo Corp., Irvine, CA, USA) as the index test, against a reference standard of laboratory assessed Hb by a SYSMEX XN-330 automated hematology analyzer (Sysmex Corp., Kobe, Japan). It was nested within the PREgnancy Care Integrating translational Science Everywhere (PRECISE) study, a prospective multi-country observational cohort study evaluating placental disorders in sub-Saharan Africa (25).

### 2.2 Study setting

Samples and data were taken from participants recruited in Kilifi County, coastal Kenya, at two public health facilities: the rural Rabai Sub-County Hospital (formerly Rabai Health Centre), and the urban Mariakani Sub-County Hospital. Both are public level three facilities managed by the Kilifi County Department of Health and Sanitation Services. The facilities provide primary antenatal care to women in neighboring communities and receive referrals from lower-level facilities in the respective sub counties. Antenatal care attendance in Kilifi county is very high with 99.3% of pregnant women achieving at least 1 visit (26). The study area has a tropical coastal climate with high temperatures and humidity and an altitude of less than 400 meters above sea level (27). Prevalence of anemia in pregnancy is higher in the coastal region than in other parts of Kenya (6).

### 2.3 Study participants

Pregnant women aged 16 to 49 years who presented to the health facility for routine antenatal care were consecutively enrolled into the ‘unselected pregnancy’ cohort and included in this analysis. There were no exclusions due to maternal clinical condition, gestational age, occupation, and other characteristics. The PRECISE study enrolment period was from 24th June 2019 to 6th December 2022. SpHb assessments were conducted throughout the study, but laboratory FBC testing was introduced in March 2021. Records selected for this nested study were for visits between 1 May 2021 and 31 December 2022. For this study we secondarily excluded records with missing Lab Hb or SpHb values and implausible values (Hb >20 g/dl).Both tests were performed for all participants in the first and second antenatal study visits.

### 2.4 The index test

The Masimo Rad-67® Pulse CO-Oximeter® (Masimo Corp., Irvine, CA, USA) was used to measure functional oxygen saturation of arterial hemoglobin (SpO2), pulse rate (PR), and perfusion index (Pi) and to obtain a non-invasive spot-check reading of total hemoglobin concentration (SpHb®). This device provides a single/spot check reading and is not used for continuous monitoring. It uses transcutaneous spectrophotometry of red and infrared light and photoplethysmography. The SpHb measurements rely on a multiwavelength calibration equation to quantify the percentage of carbon monoxide and methemoglobin and the concentration of total hemoglobin in arterial blood. Standard operating procedures for testing were derived from the device's user manual (20) and tests were performed by trained research nurses and trained research assistants with no clinical background. The averaging time SpHb setting was maintained at the factory default of “medium”. The device was used with a rainbow SET® sensor designed to provide a maximum of 1,000 tests after which no further readings would be produced, and the sensor replaced. The sensor was placed on a suitable finger determined by measuring it with a provided caliper as recommended by the sensor manufacturer. The SpHb reading was recorded immediately in the study's electronic tablets and saved in the device for future quality checks. In some cases, the device was unable to provide a SpHb reading even after troubleshooting and no result was recorded in the study database. Precautions were taken to ensure accurate measurements, including minimizing motion during measurements, and shielding the sensor from excessive light. Cable integrity and spot-check availability were regularly assessed and sensors replaced as needed.

### 2.5 The reference standard test

For laboratory estimation of hemoglobin concentration (Lab Hb), venous blood was collected from the antecubital fossa vein using an evacuated needle system, placed in a 4 ml tri-potassium ethylenediaminetetraacetic (K3EDTA) tube (BD Vacutainer, Becton Dickinson, Franklin Lakes, NJ, US), inverted 8–10 times, labelled, placed in a cool box maintained at 4–8°C and immediately transported to the on-site laboratories. Sample collection and analysis were carried out by trained research laboratory technologists. All samples were analyzed using a SYSMEX XN-330 (Sysmex Corp., Kobe, Japan) automated hematology analyzer located at each laboratory (2 analyzers). The SYSMEX XN-330 analyzer employs a chemical conversion process (cyanide-free sodium lauryl sulphate) to convert hemoglobin components into lauryl-methemoglobin, followed by absorption photometry for accurate hemoglobin measurement (28). The manufacturer reported accuracy of the device against a reference standard is a correlation coefficient, r value of ≥0.90 and bias limits ±3.5% or ±0.2 g/dl. We performed mandatory daily quality control checks on both analyzers using 3 levels (low, high and normal) of Sysmex commercial control solutions. Patient samples were only run after internal quality control had passed. Calibration of the analyzers was performed annually by the local Sysmex distributor. Initial training on use of the analyzer was also conducted by the local distributor. The SpHb reading was always done before a blood draw for Lab Hb. Blood draws for Lab Hb were done during the same visit and usually within 1 h of the SpHb reading—no treatment or other intervention was given during this interval. Lab Hb analysis was also done on the same day and within 2 h of sample collection. Laboratory staff carrying out the Lab Hb test had no access to the SpHb results.

### 2.6 Statistical analysis

Descriptive analysis of key participant characteristics and hemoglobin measurements was performed with medians and interquartile ranges (IQR) reported for continuous variables and proportions for categorical variables. We excluded any records with a Lab Hb or SpHb >20 g/dl assuming this to be biologically implausible values.

The relationship between hemoglobin by the two methods was explored using a scatter diagram and the continuous agreement evaluated using Pearson's correlation coefficient. We performed linear regression and obtained the slope and y intercept with corresponding 95% confidence intervals. The differences between paired values of SpHb and Lab Hb (bias) were assessed for normality using the Shapiro-Francia W’ test. Bland-Altman analysis was performed to assess the limits of agreement between the two methods (29). The relationship between the bias and the Lab Hb (reference standard) was graphically displayed in a modified Bland-Altman plot, preferred in this case as the Lab Hb was considered the reference method (30) Due to the non-normality of the distribution of the bias, we used non parametric methods to perform Bland Altman analysis, reporting the median bias and the limits of agreement as the 2.5th and the 97.5th percentile. Confidence intervals for these were obtained by bootstrapping.

For diagnostic accuracy, hemoglobin results were classified into categorical variables using the pre-specified WHO recommended trimester-specific cutoff values for anemia in pregnancy (31). We calculated diagnostic accuracy parameters of sensitivity, specificity, positive predictive value, and negative predictive value and the corresponding 95% confidence intervals. For clinical utility of the test, our hypothesis of a minimum sensitivity and specificity of 90% and 80% respectively was drawn from other hemoglobin point of care devices that have found utility in antenatal care (32). The analytic performance of the 2 tests was evaluated against a total allowable error of ±4.19% (33).

For this retrospective analysis, we used all eligible records within the specified study period. We did not do an *a priori* sample size calculation. A general recommendation for method comparison studies is to use at least 100 subjects (34). Larger sample sizes provide narrower confidence intervals for the limits of agreement and likely expand the range over which agreement is tested, increasing the validity of the result (34).

We performed *post hoc* sensitivity analyses by repeating Bland-Altman analysis with a dataset limited to the hemoglobin range of 8–17 g/dl by laboratory assessment to align with the manufacturer recommended range (20). We also repeated Bland-Altman analysis with a dataset that had only a single record per participant (no duplicates) to remove any influence of repeated testing in the same participants.

All data analysis was conducted in STATA SE 16 (StataCorp LLC, College Station, TX, United States). Reporting followed the Standards for Reporting of Diagnostic Accuracy studies (STARD) 2015 guidelines (35). A STARD 2015 checklist is provided in the Supplementary Material 2.

## 3 Results

**Figure 1:**
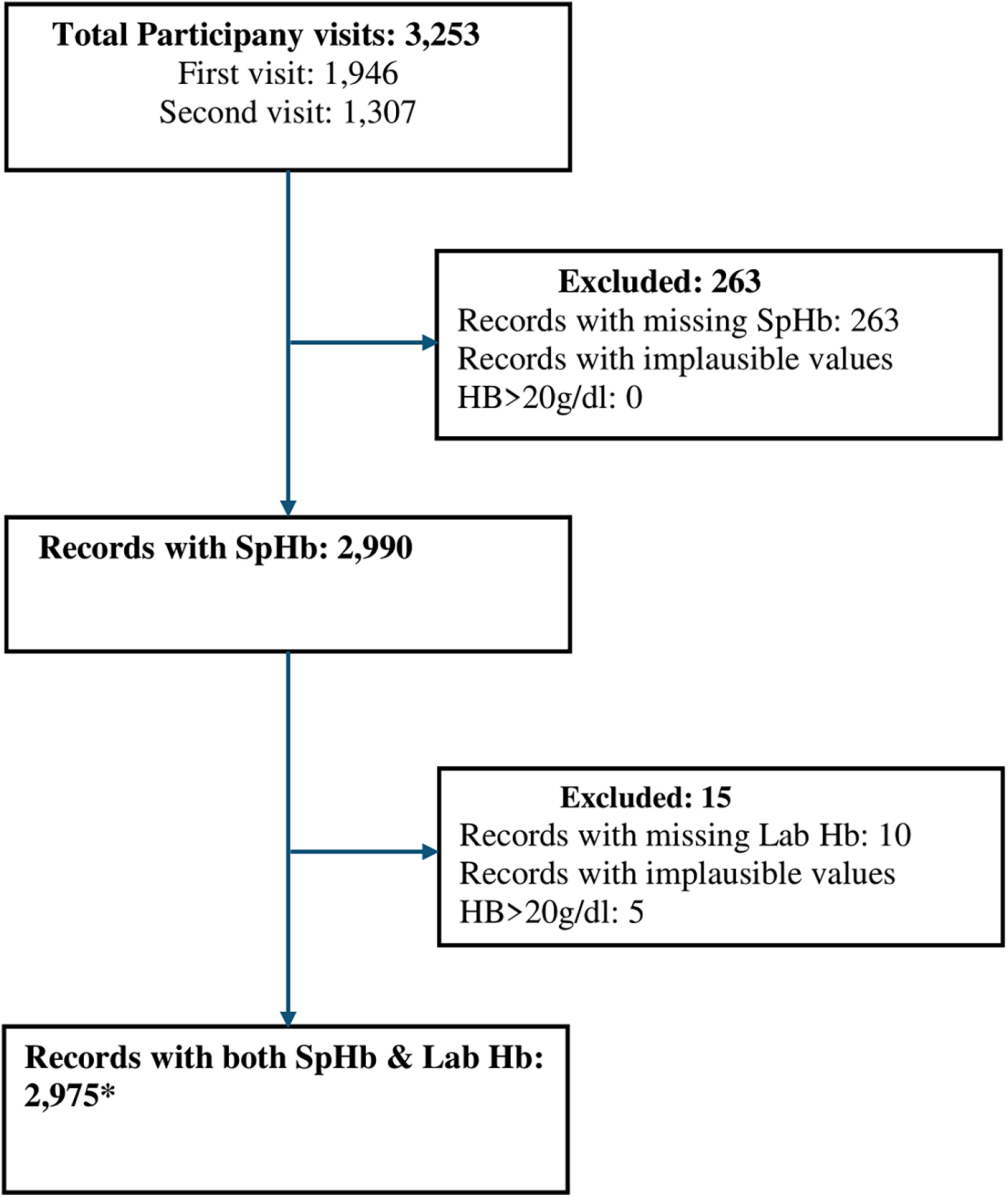
Participant flow diagram showing selection of data for analysis. *2,975 records, from 2,975 visits, from 2,203 unique study participants. SpHb- Spot-check hemoglobin concentration by Masimo Rad-67® Pulse CO-Oximeter®, Hb, haemoglobin; Lab Hb, laboratory based hemoglobin concentration by SYSMEX XN-330 automated hematology analyzer.

There was a total of 3,253 antenatal participant visits in the study period, 1,946 from the first study visit and 1,307 from the second visit. From these visits, 2,975 records that had both SpHb and Lab Hb results were selected (Figure 1). These records were from 2,203 unique participants as some participants had two study visits in the study period. The number of records that were excluded from the analysis due to missing data (missing SpHb 263, missing Lab Hb 10) or implausible values (Lab Hb >20 g/dl) were 273 constituting 8.5%.

### 3.1 Participant characteristics

The majority (35%) of study participants were aged 20 to 29 years., reflecting the young age of childbearing in the study setting. Many were in their first or second pregnancy (56%). Less than 1% reported any tobacco use. Most participants had attained only primary level education (≤8 years of formal education), and the commonest occupation was housewife. Over half of the women had body mass index in the normal range with a substantial portion overweight. Nearly all participants were of black African race. Participant characteristics are outlined in Table 1.

**Table 1:** Participant characteristics.

Participant characteristics (*n* = 2,023^a^)		
Maternal age groups in years, *n* (%)		
15–19	139	(6.87)
20–24	621	(30.70)
25–29	600	(29.66)
30–34	411	(20.32)
35–39	207	(10.23)
40–45	45	(2.22)
Maternal age in years, median (IQR)	26	(23, 31)
Parity *n* (%)		
0	574	(28.37)
1–4	1,301	(64.31)
>= 5	148	(7.32)
Gestational age at enrolment in weeks, median (IQR)	21.00	(16.29–25.43)
Gestational age at enrolment in weeks, *n* (%)		
<14	335	(16.60)
14–27	1,419	(70.32)
≥28	264	(13.08)
Missing	5	
Maternal BMI categories (kg/m^2^) *n* (%)		
Underweight, <18.5	91	(4.52)
Normal, 18.5–24.9	1,049	(52.14)
Overweight, 25.0–29.9	537	(26.69)
Obese, ≥30.0	335	(16.65)
Missing	11	
Maternal BMI, kg/m^2^, median (IQR)	24.25	(21.51, 28.02)
Tobacco use, *n* (%)		
No	2,008	(99.88)
Yes	9	(0.45)
Missing	6	
Race, *n* (%)		
Black African	2,021	(99.90%)
Other	2	(0.10)
Marital status, *n* (%)		
Single	139	(6.87)
Married/cohabiting	1,848	(91.35)
Separated/divorced	30	(1.48)
Widowed	6	(0.30)
Education level, *n* (%)		
None	183	(9.05)
Primary	1,026	(50.7)
Secondary	549	(27.13)
Higher	265	(13.10)
Occupation, *n* (%)		
Housewife	1,091	(53.98)
Self-employed/Business	403	(19.94)
Informal employment	312	(15.44)
Professional employment	146	(7.22)
Student	47	(2.33)
Other	22	(1.09)
Missing	2	
Health facility, *n* (%)		
Mariakani (urban)	1,204	(59.51)
Rabai (rural)	819	(40.49)

IQR, interquartile range; BMI, body mass index.

^a^ Characteristics assessed at first study visit in pregnancy, duplicates removed.

### 3.2 Distribution of hemoglobin values

**Figure 2:**
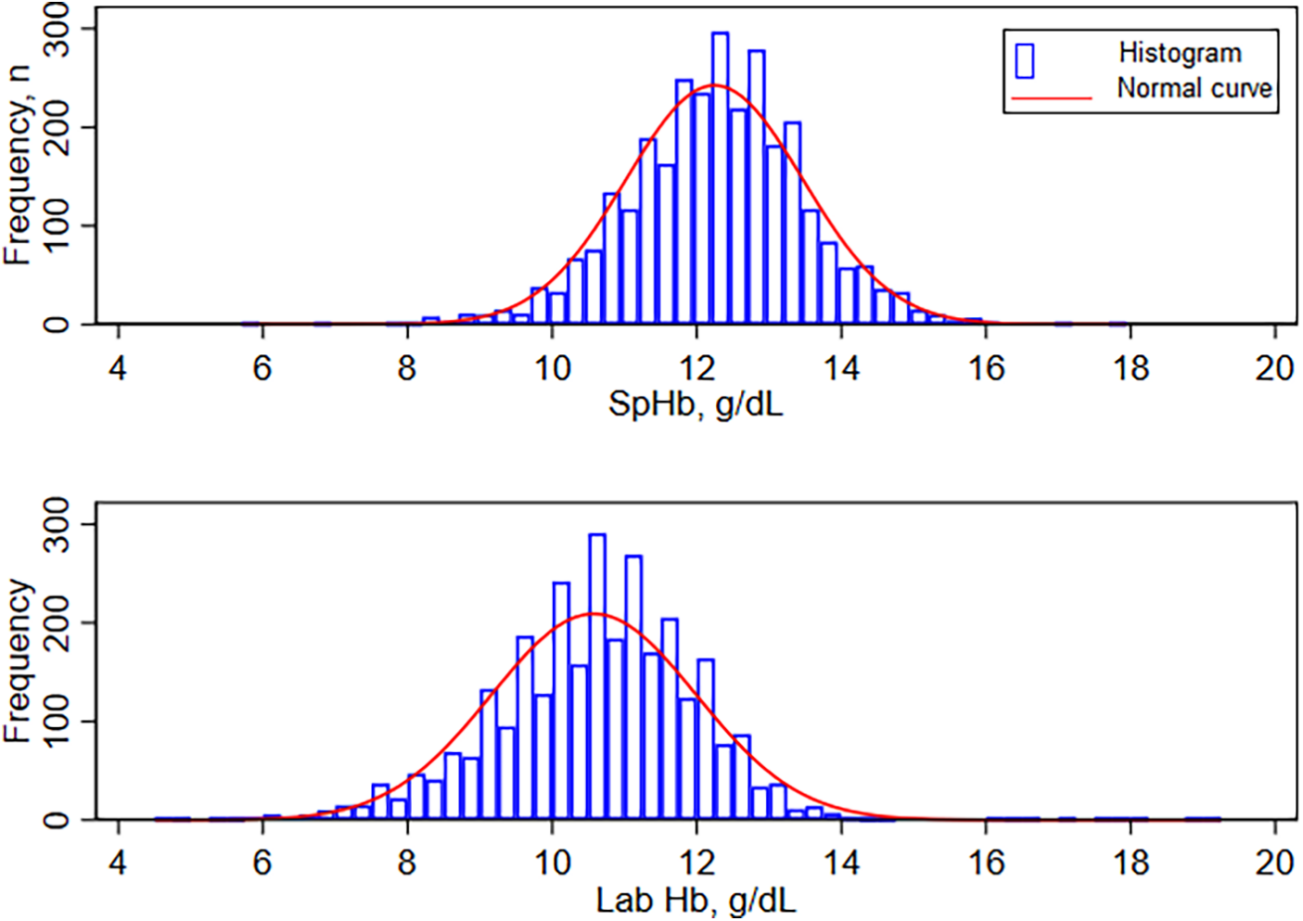
Distribution of hemoglobin by MASIMO(Rad-67) and by Sysmex XN-330. SpHb- Spot-check hemoglobin concentration by Masimo Rad-67® Pulse CO-Oximeter®, Lab Hb, laboratory based hemoglobin concentration by SYSMEX XN-330 automated hematology analyzer.

The mean SpHb by the index test for the entire dataset (*n* = 2,975) was 12.24 g/dl ± SD of 1.22, median 12.30 g/dl and IQR of 11.50–13.00 g/dl. Lab Hb results were lower with a mean of 10.58 g/dl, ±SD 1.42, median 10.70 and IQR of 9.70–11.50 g.dl. Hemoglobin results using both methods were not normally distributed (Shapiro-Francia W’ test for normal data *p* < 0.001), see Figure 2.

### 3.3 Correlation and regression

**Figure 3:**
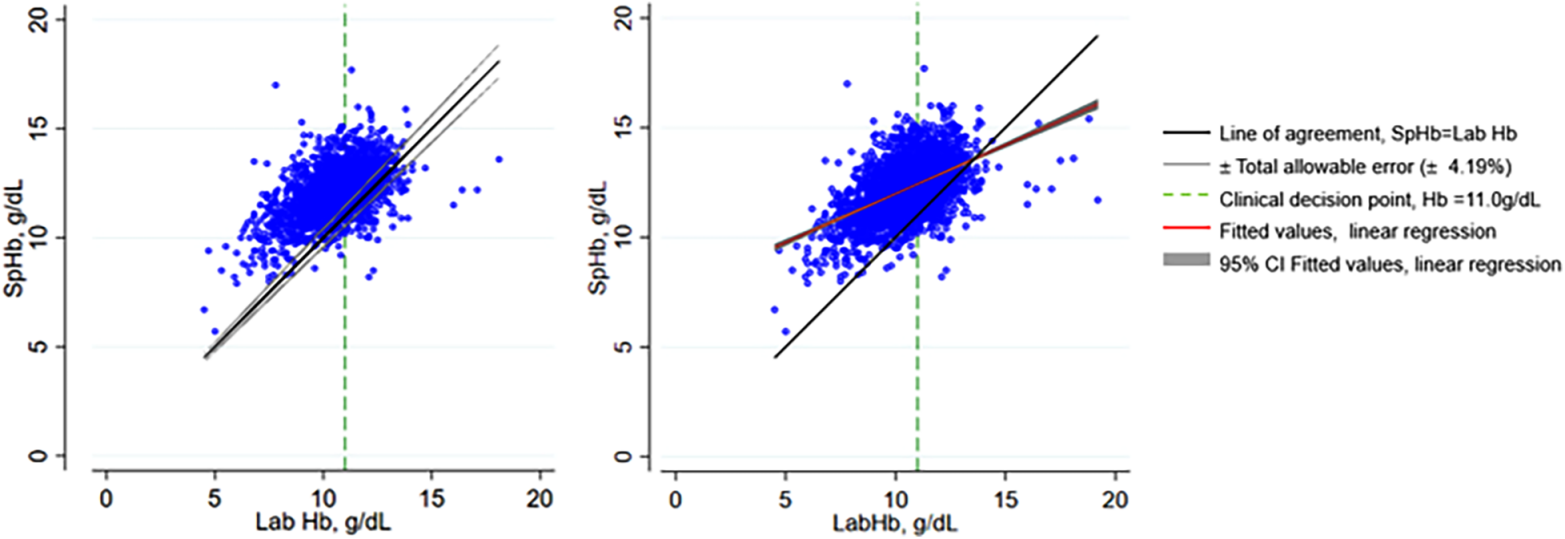
Scatter plots of SpHb and Lab Hb showing total allowable error and fitted values from linear regression. SpHb, spot-check hemoglobin concentration by Masimo Rad-67® Pulse CO-Oximeter®; Lab Hb, laboratory based hemoglobin concentration by SYSMEX XN-330 automated hematology analyzer; CI, confidence intervals.

Comparison between the 2 tests showed moderate positive correlation (r = 0.51, *n* = 2,975, *p* < 0.01). Linear regression showed a significant but weak positive slope with β of 0.44 (95% CI 0.41–0.47, *p* < 0.001)) and a y intercept at 7.59 (95% CI 7.301–7.870, *p* < 0.001), R squared of 0.26. The scatter graph with the total allowable error is shown on Figure 3A and with the fitted values from linear regression on Figure 3B.

### 3.4 Bias and Bland-Altman analysis

**Figure 4:**
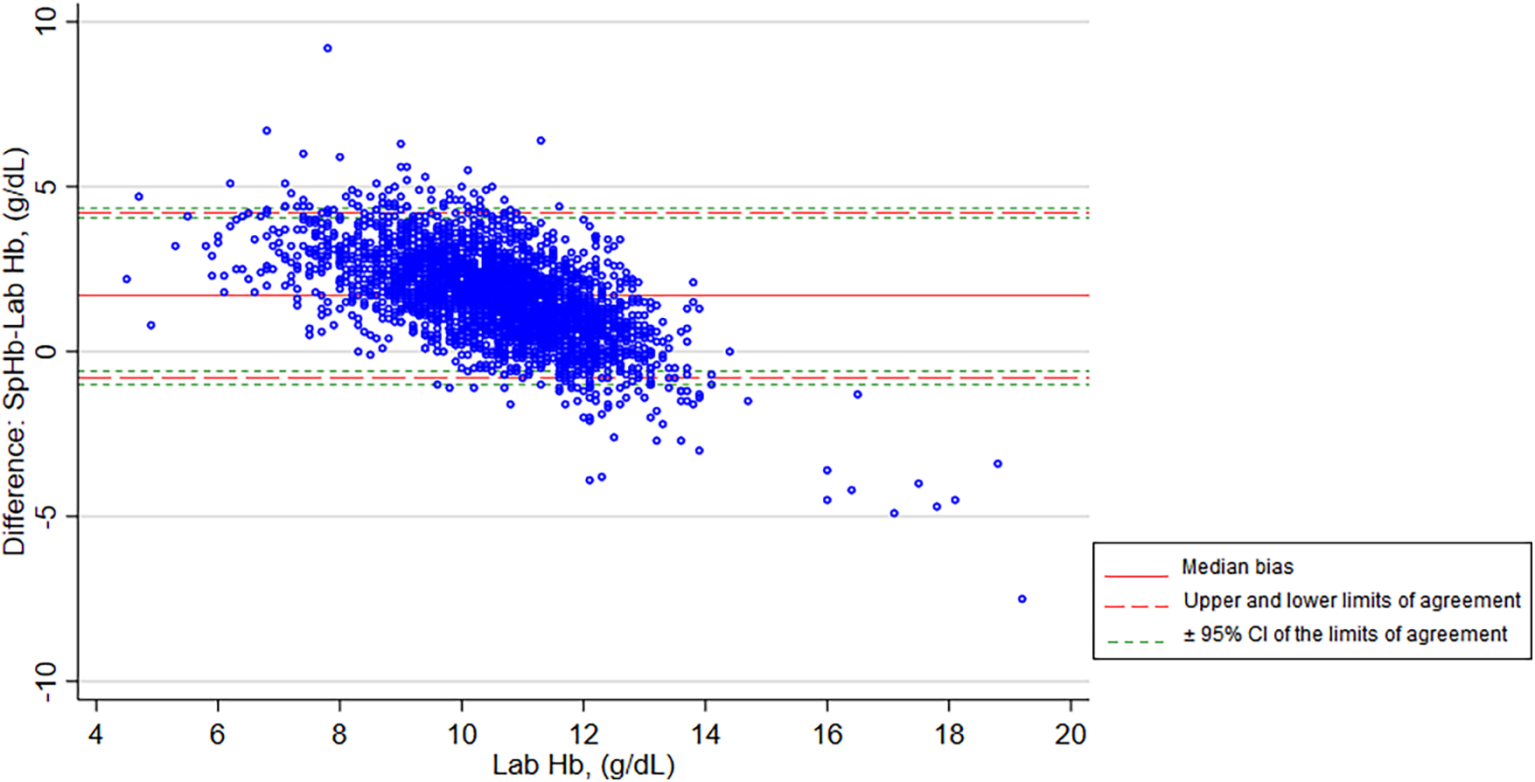
Modified Bland-Altman plot of SpHb and Lab Hb showing bias and limits of agreement. SpHb, spot-check hemoglobin concentration by Masimo Rad-67® Pulse CO-Oximeter®; Lab Hb, laboratory based hemoglobin concentration by SYSMEX XN-330 automated hematology analyzer; CI, confidence intervals.

A modified Bland-Altman plot is shown in Figure 4, with the bias plotted on the *y* axis and the Lab Hb, our reference standard, plotted on the x axis. SpHb values were likely to be higher than Lab Hb in the low hemoglobin range but lower than Lab Hb in the high hemoglobin range. The plot also shows variability dependent on the level of Hb, with greater variability away from the clinical decision point of hemoglobin = 11 g/dl. The median difference between SpHb and Lab Hb was 1.70 g/dl, with SpHb reading higher than the Lab Hb on average. The limits of agreement (2.5th and 97.5th percentiles) were −0.80 and 4.20 g/dl (Table 2).

**Table 2:** Median bias and limits of agreement from Bland-Altman analysis.

	Bias (95% CI)	Lower limit of agreement (95% CI)	Upper limit of agreement (95% CI)
Whole dataset (*n* = 2,975)	1.70 (1.65 −1.75)	−0.80 (−1.01 to −0.59)	4.20 (4.06–4.34)
Dataset with single record per participant (*n* = 2,023)	1.50 (1.41–1.59)	−1.0 (−1.22 to −0.77)	4.10 (3.94–4.26)
Dataset with Lab Hb range limited to 8 to 17 g/dl (*n* = 2,846)	1.60 (1.50 −1.70)	−0.80 (−0.98 to −0.62)	4.10 (3.96–4.24)
Dataset with single record per participant and Lab Hb range limited to 8 to 17 g/dl (*n* = 1,925)	1.50 (1.40 −1.60)	−1.00 (−1.22 to −0.78)	3.90 (3.75–4.05)

CI, confidence intervals.

### 3.5 Diagnostic accuracy

This analysis was limited to the dataset with a single record per participant (*n* = 2,023). For participants with more than 1 record, we utilized the record for the earlier visit in that pregnancy. There was a large difference between the prevalence of anemia with hemoglobin determined by the two methods, 46.37% by Lab Hb and 10.38% by SpHb. The diagnostic performance of Masimo Rad-67® Pulse CO-Oximeter®, in classifying participants to various categories of anemia is demonstrated in Table 3 and corresponding diagnostic accuracy parameters provided in Table 4. SpHb had very low sensitivity (18.66%) in detecting pregnant women with anemia and a specificity of 96.77%.

**Table 3:** The prevalence and distribution of anemia by spHb and Lab Hb.

		Lab Hb (*n* = 2,023), *n* (%)									
		Severe anemia		Moderate anemia		Mild anemia		No anemia			
SpHb (*n* = 2,023), *n* (%)	Severe anemia	2	(100.00)	0	(0.00)	0	(0.00)	0	(0.00)	2	(100.00)
	Moderate anemia	12	(23.08)	33	(63.46)	5	(9.62)	2	(3.85)	52	(100.00)
	Mild anemia	9	(5.77)	76	(48.72)	38	(24.36)	33	(21.15)	156	(100.00)
	No anemia	4	(0.22)	324	(17.87)	435	(23.99)	1,050	(57.92)	1,813	(100.00)
		27	(1.33)	433	(21.40)	478	(23.63)	1,085	(53.63)	2,023	(100.00)

Hb, hemoglobin; SpHb, spot-check hemoglobin concentration by Masimo Rad-67® Pulse CO-Oximeter®; Lab Hb, laboratory based hemoglobin concentration by SYSMEX XN-330 automated hematology analyzer.

**Table 4:** Diagnostic accuracy parameters of spHb for anemia.

	Prevalence, % (95% CI)	Sensitivity, % (95% CI)	Specificity, % (95% CI)	Positive predictive value, % (95% CI)	Negative predictive value, % (95% CI)
Anemia	46.37 (44.19–48.54)	18.66 (16.96–20.35)	96.77 (96.00–97.54)	83.33 (81.71–84.96)	57.92 (55.76–60.07)

Hb, hemoglobin; SpHb, spot-check hemoglobin concentration by Masimo Rad-67® Pulse CO-Oximeter®; CI, confidence intervals.

### 3.6 Sensitivity analysis (additional analysis)

Results of the Bland-Altman analysis comparing the two tests but limiting the dataset to records with a Lab Hb of 8 to 17 g/dl and/or limiting the dataset to 1 record per participant (*n* = 2,083) are shown in Table 2. The Bland-Altman plots are included in Supplementary Material 1. There was little difference in the bias and limits of agreement when analysis was repeated with the different datasets.

## 4 Discussion

### 4.1 Summary of main findings

There was a moderate positive correlation between SpHb and Lab Hb. Linear regression revealed that SpHb had a constant bias of 7.59 g/dl and a proportional bias where a unit increase in Lab Hb resulted in a 0.44 unit increase in SpHb which explains the positive bias in SpHb seen in the modified Bland-Altman plot at low Hb concentrations and the negative bias seen at higher Hb. The median bias between SpHb and Lab Hb was 1.70 g/dl (limits of agreement of −0.80–4.20 g/dl) which is higher than that described in previous studies using similar devices especially those assessing SpHb in pregnant women. We evaluated the potential clinical role of the test in detecting anemia in pregnancy and found that many women with anemia were wrongly classified by SpHb as “no anemia” (sensitivity of 18.66%). SpHb tended to overestimate low hemoglobin and underestimate high hemoglobin.

### 4.2 Comparison with other studies

Several studies have evaluated MASIMO devices in non-pregnant participants (adults and children). A systematic review that pooled data from studies that evaluated MASIMO Pulse co-oximeters (Rad-7 or Pronto-7), found a very small mean bias of −0.03 g/dl but wide limits of agreement −3.0 to 2.9 g/dl, and recommended caution in utilizing the devices for clinical decision making (14).

Three studies that have evaluated the devices in pregnant women have reported variable results. One study (23) found a mean negative bias of −1.09 while the other two (21, 22) reported positive mean biases of 1.33 and 1.32 respectively. All found wide limits of agreement. All these studies (21–23) were done at single health facilities and on relatively small samples (50–137 women). Our study was done at 2 health facilities and had a much larger sample size and in an area with a high prevalence of anemia. Our findings are consistent with the pregnancy studies in demonstrating wide limits of agreement (−0.80–4.20). What is unique is the bias between SpHb and Lab Hb changing with changes in hemoglobin level. None of the previous studies have recommended the use of SpHb in pregnancy.

Diagnostic accuracy parameters are known to change with prevalence of disease (36). Our study was conducted in a setting with a high prevalence of anemia (6) and this may explain the differences in diagnostic accuracy. This emphasizes the need to perform diagnostic evaluations in settings as similar as possible to where future use of the test is anticipated or planned. The unique characteristics of our participants may also explain the differences in results. Many women in our study setting participate in manual labor (e.g., farming, fetching firewood, chopping wood, and other manual domestic chores). This may affect the measurement of SpHb, perhaps through differences in skin thickness (37). Further, studies that have compared SpHb performance across different trimesters found larger bias and wider limits of agreement in the second and third trimesters (22). Most of our participants were in their second and third trimesters at the time of testing and this could explain our wide limits of agreement. Nearly all our study participants were of Black African race, but based on the manufacturers recommendation and a published study, dark skin pigmentation may not affect SpHb (38).

### 4.3 Strengths

There are various strengths to our study design. Our approach to SpHb testing as well as our choice of study site closely resembles a real-world application of SpHb testing. SpHb testing was provided at the antenatal clinic, and not at the lab, and by a research nurse, similar to the way a point of care test would be utilized if implemented into routine care. Further, our large sample size gives us more dependable results than previous studies (21–23). We enrolled unselected pregnant women who presented for routine antenatal care and used consecutive sampling with no restrictions related to maternal clinical condition, gestational age, occupation, and other characteristics. These broad inclusion criteria reflect what would be expected in a typical antenatal clinic and reduces selection bias. Though our analysis for diagnostic performance was retrospective, the main research study was prospective with all data collection and clinical procedures conducted in a standardized manner. The same laboratory reference standard was used at both sites and daily quality control checks were conducted. Neither the laboratory technologists conducting the laboratory test nor the nurses conducting the SpHb test were aware of the other's results prior to conducting their respective tests. Both tests were conducted within the same day and often less than a few hours apart with no significant clinical interventions between the two tests.

### 4.4 Limitations

As with other retrospective studies, there were some missing data: 8.5% of records were excluded due to missing SpHb or Lab Hb, or implausible values. Statistically, this is a small proportion and is unlikely to cause a significant bias. We did not document specific reasons for missed SpHb (8.1%). The pulse co-oximeter failed to provide a SpHb reading in some circumstances, and this may contribute to the number of missing SpHb data in this study. This is an important consideration for clinical application where other hemoglobin tests are not available. It is likely that some of these failed SpHb reading may have been caused by hemoglobin being out of range (20). We did not collect data on whether study participants had nail polish, hand decorations or darkened fingers due to tar at the time of SpHb determination which could potentially impact on values obtained. However, tobacco use in this study population was very low.

### 4.5 Implications for clinical practice

SpHb at the point of care can be utilized as a single test (screen and treat) or as a first of two tests with the second confirmatory test done only on those who screen positive (24). With the evidence provided in this paper, we would not recommend the use of Masimo Rad-67® Pulse CO-Oximeter® in antenatal care settings for screening of anemia using either approach. Too many pregnant women with anemia would be missed and appropriate treatment interventions for anemia would not be initiated. This is not desirable as anemia is a serious clinical condition that is associated with adverse maternal and perinatal morbidity and mortality. The prevalence of anemia in this study was very high (46.37%). The findings provide evidence from a Black African population that supports the manufacturer recommendation that it is not intended for measuring SpHb in pregnant women.

Other portable point of care devices such as HemoCue® have shown better diagnostic performance in single test evaluations (32) as well as in direct comparisons with MASIMO® Pulse CO-Oximeter® (39). The potential to use these in antenatal care should be explored further even though they lack the advantage of non-invasiveness.

### 4.6 Implications for research

There is need to further explore the diagnostic performance of and suitability for use of other non-invasive devices in antenatal care settings especially where laboratories and other hemoglobin diagnostic methods are not available.

## 5 Conclusion

We would not recommend the use of the Masimo Rad-67® Pulse CO-Oximeter® for screening or diagnosis of anemia in antenatal care settings due to very low sensitivity, a large proportional bias, and wide limits of agreement.

## Data Availability

The raw data supporting the conclusions of this article will be made available by the authors, without undue reservation.

## Group member of the PRECISE Network

Members of the PRECISE Network include: Patricia Okiro, Onesmus Wanje, Consolata Juma, Charlotte Ndana, Douglas Nyankira, Tindi Otieno, Rose Chebet, Winfred Muithi, Washingtone Owino, Quinter Okello, Michael Ochieng, Margaret Wachira, Kelvin Mbote, Mercy Osele, John Kibwanga, Happy Mdigo, Claire Ngure, Joy Wanja, Claire Kiti, Winnie Nzoka, Grace Anyange, Robin Okello, David Mwadziwe, Ruth Mambo, Sarah Maitha, Juma Gumbo, Jamal Salim, Mary Kalido, Salim Mwakanyenze, Anne Mutua, Asha Tunje, Noveline Khatievi, Nathan Barreh, Belinder Orero, Mwanajuma Khamisi, Eric Mataza, Said Lele, Olivia Kasuu, Susan Sidi, Hassan Kopa, Faith Pola, Umberto D’Alessandro, Anna Roca, Hawanatu Jah, Andrew Prentice, Melisa Martinez-Alvarez, Brahima Diallo, Abdul Sesay, Sambou Suso, Yahaya Idris, Baboucarr Njie, Fatima Touray, Fatoumata Kongira, Modou F.S. Ndure, Gibril Gabbidon, Lawrence Gibba, Abdoulie Bah, Yorro Bah, Esperança Sevene, Anifa Vala, Sonia Maculuve, Corssino Tchavana, Helena Boene, Lazaro Quimice, Salesio Macuacua, Carla Carrilho, Laura A. Magee, Marie-Laure Volvert, Thomas Mendy, Donna Russell, Prestige Tatenda Makanga, Liberty Makacha, Reason Mlambo, Lucilla Poston, Rachel Tribe, Sophie Moore, Tatiana Salisbury, Aris Papageorghiou, Alison Noble, Hannah Blencowe, Veronique Filippi, Joy Lawn, Matt Silver, Joseph Akuze, Ursula Gazeley, Judith Cartwright, Guy Whitley, Sanjeev Krishna, Marianne Vidler, Jing (Larry) Li, Jeff Bone, Mai-Lei (Maggie) Woo Kinshella, Domena Tu, Ash Sandhu, Kelly Pickerill, Carla Carillho, Benjamin Barratt.
